# Nurses' Grievances at Glasgow

**Published:** 1891-10-31

**Authors:** 


### The Hospital

A Weekly Journal of

**Science, Medicine, Nursing, and Philanthropy.**

*For Hospitals, Asylums, Infirmaries, Dispensaries, Medical Practitioners, Students, Nurses, and the Charitable Public.*

Vol. XI.—No. 266.] [Oct. 31, 1891.

### NURSES' GRIEVANCES AT GLASGOW.

To tell the story of a domestic quarrel is hardly a course which approves itself generally to sober wisdom. Sometimes, however, even a record of household strife may be so used as to make it of service in the prevention of future strife. No hospital lives to itself, any more than does an individual man or woman. It may do all hospitals, all nurses, and all managers good to devote a few minutes to the consideration of the domestic affairs of the Glasgow Royal Infirmary as they have transpired during the last few months. The institution, which is well known to the present writer, is one of the largest general hospitals in Great Britain, and it has associated with it an ancient and successful medical school. Twenty years ago it was the only general hospital available for the four or five hundred medical students gathered together at Glasgow, both those who were members of the University, and those who studied at Anderson's College, which was then called Anderson's University.

The magnitude of the operations carried on at the Infirmary will be perceived when it is stated that the institution contains five hundred and eighty-two beds for patients, which beds are mostly full, and that it employs in the care of those patients about two hundred officials and servants. Of the two hundred no fewer than a hundred and seven are nurses, and of these thirty-one are day nurses, thirty-one night nurses, and thirty-three probationers, the remaining twelve being extra nurses and extra probationers. There is thus always one nurse for every five or six patients.

Now, it is manifest that a regiment of a hundred and seven young women of the nursing class will contain a considerable variety of personal character. The post of Matron at an institution where so many are employed can be no sinecure. The Committee of Management, which the nurses look upon as their governing body, has no holiday task to perform if it is to keep its female regiment in orderly efficiency, and to satisfy the critical minds of the young women at the same time. It is to be feared that if the ears of the members of the Committee tingle at every disparaging word which is said of them behind their backs by the sharper-tongued nurses, the tingling will be practically constant, both night and day. The present writer gratefully blesses his stars in that he is neither the Matron, nor the Chairman of Committee at the Glasgow Royal Infirmary.

There has been a kind of a small Vesuvian eruption at Glasgow about the hospital. Some months ago Probationer Nurse Hunter—is she a relation, by the way, of Mrs. Robert Hunter who made war upon Miss Lückes at the London Hospital so recently?—wrote to the *North British Daily Mail* making certain complaints against the domestic management of the Infirmary. The *Daily Mail* is radical in excelsis, and was no doubt very glad to have an opportunity of making an onslaught upon any kind of constituted authority. At any rate the fire thus kindled soon spread, not only among the nurses inside the Hospital, but amongst the public outside, and the conflagration rapidly became general. The nurses were ill-fed and overworked, it was said. Their food was monotonous, it was badly cooked, the meat was too fat, the fish was sometimes putrid, and always boiled. There were no scones for supper, and what can a Scotch young woman do if she is deprived of her soda scones? Better deprive her of her sweetheart or of the Shorter Catechism. Worse than all, there was no jam! The Glasgow girls may live without scones, or catechisms, or even short-bread, but to ask them to exist without jam is to make life not worth living.

Of course when all these things came to the public ears, and when, above all, the empty jam pot was raised aloft on the standard of Scottish patriotism, it was evident that either the world must come to an end, or the managers of the hospital must deal promptly with the alarming situation. With all speed, therefore, a special committee was appointed to enquire into the circumstances of the alleged grievances, and if it should be found possible, to devise measures for the satisfaction of the nurses. All this was quite as it should be. The enquiry commission, consisting of some of the most eminent citizens and medical men of Glasgow, met for the prosecution of their investigation on the third of September, and continued their sittings with occasional intermissions for two or three weeks. Their enquiry was of the fullest and most exhaustive kind: every nurse, every resident medical officer, every official of every kind who had a shadow of complaint to make was invited to make it with the utmost possible fulness and freedom, and without fear of consequences.

The published report of the Investigation Committee is now before us; and one is inclined to say, with the apostle James, "Behold, how great a matter a little fire kindleth." On the one side, nurse after nurse spoke against almost every article of food which had come to the table, though there were some nurses who reported more favourably of it. On the other side the resident medical officers, the patients, and certain other officials, whose food was supplied from the same stores and cooked and served by the same cooks, had practically no complaints to make at all. Moreover, on three separate occasions several members of the Investigation Committee took the opportunity, of course, without giving the cook or any other person previous notice, of dropping in at the dinner-hour and taking dinner with the nurses. On all those occasions the members of the Committee declared the dinners to be excellent; and in this all the nurses themselves concurred, with only one exception. This young lady expressed the opinion that the Sunday plum tart was not equal to the plum tart of her girlish dreams. Here, then, was a conflict of evidence; but taking into consideration the fact that on the one hand the resident medical officers, the patients, and the members of the Investigation Committee found the food excellent, and that on the other hand a certain number of the nurses themselves had little fault to find with it, whilst those nurses who did find fault, to a great extent merely repeated each other's complaints, one cannot but conclude that probationer nurse Hunter has made a great deal out of a very little; and that if the nurses themselves had been as loyal to their hospital as they should have been, the whole affair could have been settled to their entire satisfaction without disturbance of the peace, and without publicity of any kind.

The question of long hours was similarly investigated, and with like results. It was found that certain irregularities had crept in which pressed hardly on some of the nurses, but for which, oddly enough, the nurses themselves were principally responsible. The Committee have acted throughout with great kindness and discretion. They have recognised the important fact that they were dealing with women and not with men; and that women have fastidious appetites, critical tempers, and sometimes gossiping tongues. They have, therefore, like men of sense, recommended certain modifications in the working hours and in the hospital dietary. There is to be greater variety, alike in the meat and in the cooking of it. The plum tarts are to be raised to a higher standard of perfection, the scones, it is to be hoped, will in future be light and new, and the jam-pot, beloved of probationers, is to be like the widows' cruise of oil, unfailing and of the sweetest brand.

Truth to say, it is impossible to help being a little amused that a large institution like the Glasgow Royal Infirmary, consisting of nearly a thousand persons in residence, should have been set by the ears and made a mark of public approbium for so very small a matter. One would like to urge upon nurses everywhere, probationers in particular, that they are not children any longer, but women. They owe it to themselves, therefore, to behave with self respect and dignity, and not to go gossiping and whining about like Cockney "slaveys" or the stockingless denizens of the Salt Market. Besides, do they not owe some loyalty to the hospital which is training them, and which is to be their permanent alma mater? No doubt things may go wrong in hospitals just as in homes and elsewhere. But when any complaint needs to be made it should be made first of all to the proper authorities. The very last thing to be done is to rush with it to the public newspapers. If the authorities refuse to be reasonable, then the editors and the public may be called in to denounce or arbitrate. But every nurse and probationer in Glasgow, and, indeed, in every other hospital, will do well to remember that, as the Scotch proverb says, it is generally "an ill bird that fouls its own nest."

